# Heritability of sleep architecture based on home polysomnography

**DOI:** 10.1111/jsr.14448

**Published:** 2024-12-29

**Authors:** Mario Leocadio‐Miguel, Tâmara P. Taporoski, Felipe Beijamini, Francieli S. Ruiz, Andrea R. V. R. Horimoto, Alexandre C. Pereira, Kristen L. Knutson, Malcolm von Schantz

**Affiliations:** ^1^ Faculty of Health and Life Sciences Northumbria University Newcastle‐upon‐Tyne UK; ^2^ Northwestern University Feinberg School of Medicine Chicago Illinois USA; ^3^ Harvard Center for Population and Development Studies Harvard University Cambridge Massachusetts USA; ^4^ Federal University of Fronteira Sul Realeza Brazil; ^5^ Brigham and Women's Hospital Harvard Medical School Boston Massachusetts USA; ^6^ Incor, University of São Paulo School of Medicine São Paulo Brazil

**Keywords:** electroencephalogram, genetic admixture, genetic variance, paradoxical sleep, slow‐wave sleep

## Abstract

We aimed to establish the heritability of polysomnography measures through the analysis of home polysomnography recordings from 648 participants in the Baependi Heart Study, a rural, family‐based, genetically admixed cohort based in the southeast of Brazil. Sleep polysomnography staging variables were computed, and narrow‐sense heritability values were derived. The heritability (*h* ^2^) of polysomnography total sleep time was 0.18 ± 0.08 (*p* = 0.007). Including age and sex did not change the heritability estimates of the model. Wake after sleep onset did not show significant heritability (*h* ^2^ = 0.02 ± 0.07, *p* = 0.39). The unadjusted model for N1 resulted in a heritability estimate of 0.22 ± 0.10 (*p* < 0.003), and of 0.26 ± 0.10 (*p* = 0.003) in the adjusted model. Time spent in N2 had an unadjusted heritability of 0.18 ± 0.09 (*p* = 0.01) and adjusted *h* ^2^ of 0.22 ± 0.10 (*p* = 0.007). The heritability of the total time spent in N3 was 0.35 ± 0.09 (*p* < 0.001) in the unadjusted and 0.38 ± 0.09 (*p* < 0.001) when sex, age and age*age were considered in the model. By contrast, no variance in the total time spent in rapid eye movement could be significantly attributed to genetic variance. In terms of the heritability of the apnea–hypopnea index, 17% of its variance could be attributed to genetic factors (0.17 ± 0.08, *p* = 0.02). This is the first report of heritability of electroencephalographic‐derived sleep parameters from a larger population sample, and the first one performed in a population with a majority above 25 years of age. Our findings indicate the potential feasibility of future genome‐wide association studies of non‐rapid eye movement sleep stages in pooled population samples.

## 1 INTRODUCTION

The gold‐standard method for assessing sleep structure is polysomnography (PSG), a technique derived from the development of the electroencephalogram (EEG) in the late 1920s. Seminal work in the 1930s demonstrated that not only do human EEG signals have a reconcilable individual pattern (Travis & Gottlober, 1936), but they also present intra‐individual stability over time (Travis & Gottlober, 1937) and are heritable, as originally demonstrated with a waking EEG measure in a small twin‐based study (Davis & Davis, 1936). Together with the report of a higher concordance in the sleep habits of monozygotic (MZ) twins than in dizygotic (DZ) twins in 1937 by Geyer (1937), these early studies paved the way for research into the heritability of sleep characteristics.

Despite the substantial heterogeneity in sleep duration measures obtained through self‐reports, actigraphy and PSG, combined meta‐analysis suggests that approximately 40% of the variability in sleep duration is genetically determined (Kocevska et al., 2021; Madrid‐Valero et al., 2020). When only PSG measures of sleep duration are taken into account, this percentage drops to 27%, which is consistent with the fact that most PSG protocols constrain sleep time to some degree (Kocevska et al., 2021). Previous studies of the heritability of other PSG measures, such as the duration of sleep stages, analysed data from MZ and DZ twin pairs, like the pioneering study of Geyer (Geyer, 1937). Their common features are that they are based on relatively small numbers of participants, which limits the ability to control for covariates. Linkowski (1999) reported on a series of experiments based on the Brussels Study, investigating healthy Belgian twins between 1982 and 1990. They significantly improved the methodological approach in PSG twin studies (26 pairs of MZ and DZ twins in each study) by collecting sleep data from consecutive nights while accounting for the effects of cohabitation and total sleep time (TST). Although, like most older publications on heritability and sleep architecture, it did not use Falconer's formula (Falconer & Mackay, 2009) to compute *h* ^2^ which makes a direct comparison to our data more difficult, it concluded that genetic influences substantially determine the variance in the duration of stage 2 and slow‐wave sleep (Linkowski et al., 1989, 1991). Later, Ambrosius et al. conducted a study comparing the sleep characteristics of 49 pairs of young adult German twins (mean age 23.4 years). PSG recordings suggested substantial genetic components to the duration of sleep Stage 3 (N3) and rapid eye movement (REM) sleep (Ambrosius et al., 2008). A reanalysis of the dataset previously published by Ambrosius et al. and focusing on REM sleep microstructure found a significant genetic influence on the amount of REM sleep and the REM power spectrum (Adamczyk et al., 2015).

However, despite still being considered by many as the gold‐standard for estimating heritability, twin studies are not free from limitations. There is a historic lack of geographical and ancestral diversity in twin samples used to estimate heritability in favour of populations of European heritage (Friedman et al., 2021). Sample sizes are generally limited and age range narrow. Most published heritability studies are derived from twin samples, which contain specific genetic variance information, and the equal environment assumption of the classical twin models potentially leads to inflated heritability estimates (Mayhew & Meyre, 2017). By contrast, pedigree‐based studies of extended families are a valuable tool for determining the extent to which genetic factors contribute to phenotypic variation among larger numbers of individuals and age ranges, composing a higher genetic variance, as well as for comparing data with modern registry‐based genetic studies (Almasy, 2022). The current study capitalized on the unique combination of a family‐based cohort study with the availability of home PSG recordings and a wide age range.

## 2 METHODS

### 2.1 Sample

This sample was derived from the sleep ancillary study to the Baependi Heart Study (BHS), a family‐based cohort study based in a rural town in the state of Minas Gerais in the South‐East of Brazil. The town has limited inbound migration, and the residents have a high degree of admixture between European, African and Native American ancestries. The methodology for recruitment has been described in detail previously (Egan et al., 2016). Due to its reduced inbound migration, cohesive culture and lifestyle, this sample has proven useful for estimating the heritability of several phenotypes (de Oliveira et al., 2008; Egan et al., 2016; Evans et al., 2021; Leite et al., 2020; Leocadio‐Miguel et al., 2021; Taporoski et al., 2019). The collection and processing of data from the BHS is ongoing. The subsample used here includes participants whose data and relationship to the kinship matrix were collected and processed until November 2022. The protocol for this study conformed to international ethics standards based on the Declaration of Helsinki. It was approved by the local ethics committee (Hospital das Clínicas, University of São Paulo, Brazil, number 0494/10). Each volunteer provided informed consent before participation. The specific inclusion criteria for the present analysis include having completed a full‐night ambulatory PSG, and being mapped to at least one of the family pedigrees of the study. From the original sample of the sleep ancillary study, 684 participants met the criteria and were included in the analysis. The sample was derived from 101 family pedigrees with a median of three individuals from each one.

### 2.2 Measures

The participants underwent 1 night of type II at‐home PSG. Following the setup recommended by the American Academy of Sleep Medicine (AASM) (Iber et al., 2007), the PSG recordings included the following: six EEG channels (F3, F4, C3, C4, Cz, O1, O2) and two auricular references (A1, A2), left and right electrooculographic electrodes, two electromyographic electrodes were used, both were placed underneath the chin 2 cm away from the mandible on either side. Two trained sleep technologists staged the PSG recordings according to the AASM sleep scoring criteria (Iber et al., 2007). The following characteristics of sleep were calculated: TST; total time in non‐REM (NREM) sleep 1 (N1); total time in NREM sleep 2 (N2); total time in NREM sleep 3 (N3); total time in REM sleep (REM); wake after sleep onset (WASO). The respective percentages of each sleep stage were also calculated.

We used the apnea–hypopnea index (AHI) as an indicator of breathing‐related sleep disorder. The AHI was based on the average number of apneas and hypopneas per hour of sleep. Hypopneas were characterized by peak signal excursions that dropped by ≥ 30%, lasting ≥ 10 s and with an oxygen desaturation of ≥ 3% or the event was associated with arousal (AASM definition 1A; Troester et al., 2023).

### 2.3 Statistical analysis

Descriptive statistics were used to both characterize the study sample and describe central tendency (mean ± standard deviation). Sex differences were examined using the Mann–Whitney test, with the effect size given by the rank biserial correlation. Spearman's rho correlation test was used to explore the associations between age and each variable; *p*‐values < 0.05 were considered significant.

Narrow‐sense heritability (h2) was computed for each sleep characteristic (total time and % of TST) and was obtained through the maximum likelihood estimate‐based variance components approach using the Sequential Oligogenic Linkage Analysis Routines (SOLAR Eclipse version 9.0.1 for Linux) (Almasy & Blangero, 1998). In the variance component model, the trait level $\gamma_{i}$ for the individual i is described by:

$$\gamma_{i}=\mu +\sum_{j=1}^{c}\beta_{j}X_{\mathrm{ij}}+g_{i}+e_{i},$$

where μ is the mean of the trait, and $\beta_{j}$ represents the coefficient for the covariate j, which assumes the value $X_{\mathrm{ij}}$ for the individual i. The remaining parameters $g_{i}$ and $e_{i}$ represent the random polygenic genetic and error components, respectively, and are assumed to be uncorrelated and normally distributed with mean zero and variance $\sigma_{a}^{2}$ and $\sigma_{e}^{2}$.

Heritability estimate (*h* ^2^) is the ratio of the variance of each trait explained by the additive polygenic effects to the total variance for the individual measure. In a narrow sense approach, the heritability of each sleep characteristic was represented by the proportion of the phenotypic variance attributable to additive genetic effects and is given by *h* ^2^ = $\sigma_{a}^{2}/\sigma_{p}^{2}$, where $\sigma_{a}^{2}$ is the variance due to the additive effects of genes, and $\sigma_{p}^{2}$ is the phenotypic variance. The overall phenotypic variance was estimated from the observed distribution of each sleep characteristic in the sample. It was partitioned into genetic and environmental components using the observed covariance among family members, as $\Omega =2\Phi \sigma_{a}^{2}+I\sigma_{e}^{2}$, where Ω is an “*n* × *n*” matrix of the “*n*” individuals in the data set, 2Φ is the structuring matrix of the coefficient of relationship, and “*I*” is an identity matrix that represents the structuring matrix for $\sigma_{e}^{2}$, the variance due to residual environmental factors. Estimates of the mean and variance components were obtained using maximum likelihood methods (Almasy & Blangero, 2010).

In other words, heritability was calculated by decomposing the overall variance of each sleep variable into its genetic and environmental sources, based on the covariance of the phenotypes among family members and their degree of relatedness, while adjusting for potential confounders. The heritability value ranges from 0 to 1, in which 0 indicates no genetic contribution and 1 indicates that genetics entirely determines the trait. When the genetic contribution is not significant in the heritability calculation, there is no significant benefit in considering kinship variance in the model. However, when the heritability model is significant, it indicates that there is an important source of variation attributed to genetic factors.

All variables not within the normal distribution range were submitted to the inverse normalized transformation command in SOLAR to reach normality parameters. We calculated the heritability of all continuous variables using both unadjusted and adjusted models for covariates. The best‐fitting model was obtained through a stepwise approach, including sex, age, age*sex, age*age and TST as covariates. For each trait, three models were fitted to the data: (1) unadjusted, which did not include any fixed effects; (2) best adjusted fitting model, including fixed effects; (3) including the AHI as a covariate; and (4) with the further addition of body mass index (BMI) as a covariate. The best model refers to the final model where only significant covariates were kept as fixed effects.

## 3 RESULTS

The characteristics of the 684 participants are summarized in Table 1. The majority of participants were female (62.9%). The mean age was 49.9 ± 13.6 years, ranging from 18 to 89 years.

**TABLE 1:** Descriptive characteristics of the sample and sleep characteristics evaluated with PSG.

Characteristic	Mean ± SD (*n* = 684)	Minimum	Maximum
Age (years)	49.9 ± 13.6	18	89
TST (hr)	6.5 ± 1.0	4.0	9.9
WASO (min)	61 ± 43	0	254
N1 (min)	50 ± 32	3	292
N2 (min)	221 ± 53	2	459
N3 (min)	43 ± 34	0	230
REM (min)	77 ± 31	0	171
N1%	13 ± 9	0.6	92.7
N2%	56 ± 10	0.6	97.9
N3%	11 ± 9	0	62.0
REM %	20 ± 7	0	41.6
AHI	12.2 ± 17.8	0	130.3
BMI	27.0 ± 5.2	16.0	51.5

AHI, apnea–hypopnea index; BMI, body mass index; REM, rapid eye movement; TST, total sleep time; WASO, wake after sleep onset.

Age was positively associated with the total duration of N1 (rho = 0.26, *p* < 0.001), AHI (rho = 0.42, *p* < 0.001) and the duration of WASO (rho = 0.38, *p* < 0.001). The opposite was found for the TST (rho = −0.23, *p* < 0.001), the total duration of N3 (rho = −0.29, *p* < 0.001) and the total duration of REM (rho = −0.32, *p* < 0.001), which all decreased as a function of age.

We observed a significant effect of sex on the duration of N1 and N3, as well as in AHI and BMI. The total duration of N1 was 59 ± 35 min for male and 46 ± 28 min for female participants (*U* = 66,900, *p* < 0.001, rank‐biserial correlation = 0.28). By contrast, the total duration of N3 was significantly lower (31 ± 29 min) in males when compared with female participants (47 ± 35 min; *U* = 39,584, *p* < 0.001, rank‐biserial correlation = −0.27). Moreover, male participants presented higher levels of AHI than females (14.7 ± 18.4 versus 12 ± 17.8, respectively; *U* = 63,243, *p* < 0.001, rank‐biserial correlation = 0.16). Finally, female participants exhibited a higher BMI, when compared with males (*U* = 47,050, *p* = 0.004, rank‐biserial correlation = −0.13). No significant differences in other sleep characteristics were found between the sexes.

Table 2 shows that the heritability of the TST was 0.18 ± 0.08 (*p* = 0.007). In other words, 18% of the variance of TST could be attributed to the genetic variance. The inclusion of age, sex, AHI and BMI did not improve the adjustment of the model. By contrast, WASO did not show significant heritability in our sample (*h* ^2^ = 0.02 ± 0.08, *p* = 0.39 in the best‐fitting model).

**TABLE 2:** Heritability (*h* ^2^) estimates for sleep characteristics, adjusted for covariates.

Trait	Model	*h* ^2^ ± SE	CI	*p*‐Value	*p*‐Value of covariates	Variance explained	$\sigma_{g}^{2}$	$\sigma_{e}^{2}$
TST	Unadjusted	0.18 ± 0.08	0.02–0.33	0.007			0.2	0.91
	Adjusted	0.14 ± 0.07	0.00–0.27	0.02	Age 0.005)	0.05	0.15	0.91
	+ AHI	0.13 ± 0.08	−0.02 to 0.27	0.03	Age (< 0.001), AHI (0.09)	0.05	0.14	0.91
	+ BMI	0.10 ± 0.08	−0.05 to 0.25	0.07	Age (< 0.001), BMI (0.04)	0.05	0.11	0.93
WASO	Unadjusted	0.02 ± 0.08	−0.11 to 0.15	0.39			0.02	0.88
	Adjusted	000	—	0.5	Age (< 0.001), Sex (0.04), Age*Sex (0.06), TST (< 0.001)	0.2	0.0005	0.72
	+ AHI	0.00	—	0.5	Age (< 0.001), Sex (0.08), Age*Sex (0.03), AHI (< 0.001), TST (< 0.001)	0.22	4E‐13	0.7
	+ BMI	0.00	—	0.5	Age (< 0.001), Sex (0.08), Age*Sex (0.03), AHI (< 0.001), TST (< 0.001)	0.22	1E‐16	0.7
N1 (min)	Unadjusted	0.22 ± 0.10	0.06–0.45	0.005			0.21	0.74
	Adjusted	0.26 ± 0.10	0.07–0.46	0.003	Age (< 0.001), Sex (< 0.001), TST (< 0.001)	0.15	0.21	0.61
	+ AHI	0.23 ± 0.10	0.05–0.44	0.007	AHI (< 0.001), Sex (< 0.001), Age (< 0.001), TST (< 0.001)	0.25	0.17	0.55
	+ BMI	0.23 ± 0.10	0.02–0.41	0.006	AHI (< 0.001), Sex (< 0.001), Age (< 0.001), TST (< 0.001), BMI (0.06)	0.25	0.17	0.55
N2 (min)	Unadjusted	0.18 ± 0.09	0.03–0.38	0.01			0.18	0.81
	Adjusted	0.22 ± 0.10	0.02–0.41	0.007	Age*Age (0.09), TST (< 0.001)	0.5	0.11	0.38
	+ AHI	0.22 ± 0.10	0.02–0.33	0.007	Age*Age (0.06), TST (< 0.001)	0.5	0.11	0.38
	+ BMI	0.24 ± 0.10	0.00–0.31	0.004	Age*Age (0.1), TST (< 0.001), BMI (0.009)	0.5	0.12	0.37
N3 (min)	Unadjusted	0.35 ± 0.09	0.19–0.50	< 0.001			0.32	0.61
	Adjusted	0.38 ± 0.09	0.22–0.53	< 0.001	Age (< 0.001), Sex (< 0.001), Age*Age (0.01)	0.14	0.3	0.49
	+ AHI	0.33 ± 0.09	0.13–0.48	< 0.001	Age (< 0.001), Sex (< 0.001), Age*Age (0.03), AHI (< 0.001)	0.19	0.24	0.51
	+ BMI	0.33 ± 0.09	0.13–0.48	< 0.001	Age (< 0.001), Sex (< 0.001), Age*Age (0.03), AHI (< 0.001)	0.19	0.24	0.51
REM (min)	Unadjusted	0.07 ± 0.09	−0.10 to 0.24	0.19			72.31	969.3
	Adjusted	0.13 ± 0.1	−0.05 to 0.29	0.08	Age (0.01), Age*Sex (0.02), TST (< 0.001)	0.36	83.9	575
	+ AHI	0.14 ± 0.10	−0.05 to 0.33	0.06	Age (0.02), AHI (0.06), Age*Sex (0.05), TST (< 0.001)	0.37	90.87	565.1
	+ BMI	0.14 ± 0.10	−0.03 to 0.31	0.06	Age (0.008), Age*Sex (0.08), TST (< 0.001), BMI (0.02)	0.37	89.58	564.1
AHI	Unadjusted	0.17 ± 0.08	0.00–0.31	0.02			0.16	0.78
	Adjusted	0.27 ± 0.10	0.07–0.42	0.001	Age (< 0.001), Sex (< 0.001), Age*Sex (< 0.001), Age*Age (0.01)	0.21	0.2	0.54
	+ BMI	0.31 ± 0.10	0.13–0.44	< 0.001	Age (< 0.001), Sex (< 0.001), Age*Sex 0.002), Age*Age (0.04), BMI (< 0.001)	0.26	0.21	0.48

We included the unadjusted and adjusted models for each trait, the significant covariates, the variance explained by final covariates, and the variance due to genetic $\left(\sigma_{g}^{2}\right)$ and residual environmental factors $\left(\sigma_{e}^{2}\right).$

AHI, apnea–hypopnea index; BMI, body mass index; CI, confidence interval; REM, rapid eye movement; TST, total sleep time; WASO, wake after sleep onset.

Table 2 also presents the heritability of the total time spent in N1, N2, N3 and REM sleep, respectively. The unadjusted model for N1 resulted in a heritability estimate of 0.22 ± 0.10 (*p* = 0.005). The adjusted best model, including age, sex and TST as covariates, slightly increased *h* ^2^ to 0.26 ± 0.10 (*p* = 0.003). The variance explained by the best‐fit covariates was 0.15. The addition of the AHI as a covariate resulted in a reduction of *h* ^2^ to 0.23 ± 0.10 (*p* = 0.007), and a sustained reduction was obtained with the further inclusion of BMI, *h* ^2^ = 0.23 ± 0.10 (*p* = 0.006). However, the confidence intervals (CIs) in the three models overlap substantially, so there may not be a significant reduction in the heritability estimate after adjustment for AHI or BMI. Total time spent in N2 had lower heritability than N1, as the unadjusted model resulted in an *h* ^2^ = 0.18 ± 0.09 (*p* = 0.01), and age*age + TST increased this value to 0.22 ± 0.10 (*p* = 0.007), explaining 0.5 of its variance. When AHI was considered, the *h* ^2^ estimate was similar, and remained significant (*p* = 0.007). Moreover, the addition of BMI resulted in a higher *h* ^2^ estimate, 0.24 ± 0.10 (*p* = 0.004). The estimated heritability of the total time spent in N3 was 0.35 ± 0.09 (*p* < 0.001) in the unadjusted model, and the inclusion of age, sex and age*sex as covariates raised this value to 0.38 ± 0.09 (*p* < 0.001) in the adjusted best model. The variance explained by the final covariates was 0.14. The inclusion of the AHI among the covariates reduced *h* ^2^ to 0.33 ± 0.09 (*p* < 0.001), and remained the same with the further addition of BMI in the model. However, again, the CIs in the three models overlap substantially, so this reduced estimate may not be significantly different. By contrast, no significant variance in the total time spent in REM sleep could be attributed to genetic variance as the estimate of heritability of REM was not significant, *h* ^2^ = 0.07 ± 0.09 (*p* = 0.19) in the unadjusted model, as well as in the adjusted that included age, TST and age*sex as covariates (*h* ^2^ = 0.13 ± 0.1, *p* = 0.08). Accounting for AHI changed the heritability of the REM sleep duration to 0.14 ± 0.10, remaining below the statistical significance threshold (*p* = 0.06). Importantly, the inclusion of TST as a covariate did not change the fitness of the adjusted models for any other sleep stage.

We observed a significant estimated heritability of the AHI. According to the unadjusted model, 17% of the variance of AHI could be attributed to genetic factors (0.17 ± 0.08, *p* = 0.02). When age, sex and their interaction were included as covariates, the heritability estimates increased to 0.27 ± 0.10 (*p* = 0.001). The variance explained by the final covariates was 0.21. Adjustment for BMI also impacted the heritability estimate of AHI, increasing it to 0.31 ± 0.10 (*p* < 0.001).

## 4 DISCUSSION

This study investigated the heritability of sleep structure in a family‐based cohort, and determined heritability estimates of total sleep duration as well as the duration of sleep stages. We observed significant heritability in the duration of all NREM sleep stages and TST, but not in REM sleep or WASO.

Our results show that genetic factors account for a significant proportion of the phenotypic variance in the duration of NREM sleep stages, which substantiates previous reports from twin studies. Our findings highlight the high heritability of NREM sleep. This has previously been reported in a small study of 20 twin pairs (De Gennaro et al., 2008). Our study confirms and validates with an extended‐family design and a 16‐fold larger sample size. Compared with the other NREM stages, the duration of stage N3 sleep achieved the highest heritability estimate in our study (38%). According to the two‐process model of sleep regulation, the interaction between circadian process C and homeostatic process S determines sleep propensity (Borbely, 1982). Under conditions of sleep deprivation, the duration of stage N3 increases as a homeostatic response, an indicator of sleep pressure, to the detriment of the duration of other sleep stages (Aeschbach et al., 1996). Interestingly, the extent to which sleep homeostatic pressure builds up in response to sleep deprivation has also been confirmed to be genetically determined in humans (Goldschmied et al., 2023).

In contrast to our findings for NREM sleep, we found no significant genetic contribution to REM sleep duration. Unlike an early report of a significant genetic contribution (Webb & Campbell, 1983), subsequent studies performed in the 1990s did not observe this (Ambrosius et al., 2008; Linkowski et al., 1989, 1991; Rusterholz et al., 2018). However, Adamczyc et al. revisited this issue and observed that genetic factors contributed to the variance in the duration of REM sleep (Adamczyk et al., 2015). It is worth mentioning that the literature on the heritability of REM sleep duration has concentrated not only on twins but specifically on young adults with an average age of no more than 25 years (Adamczyk et al., 2015; Linkowski, 1999; Webb & Campbell, 1983), while Rusterholz et al. (2018) collected data from a sample of adolescents, an age range when sleep patterns differ greatly compared with adulthood. The average age of our sample is 49.9 years and, despite the lack of specific studies focusing on how the heritability of REM sleep duration is dependent on age, this factor could contribute to explaining our results. It could be that with increasing age, REM sleep regulation may become increasingly impacted by environmental factors. A previous study with the same cohort showed that age was a significant predictor of REM length across the age span, with REM duration peaking at about 35 years and decreasing by approximately 50 years old (Taporoski et al., 2024). Moreover, the research design adopted by Adamczyk et al. included three consecutive nights of sleep in the laboratory, and only data from the second and third nights were used for the heritability estimate, accounting for the first‐night effect, which disproportionately affects REM sleep (Ding et al., 2022). In line with these results, microarousals during REM sleep were not only demonstrated as part of the first‐night effect but also showed substantial inter‐individual variability (Sforza et al., 2008). Therefore, it is possible that first‐night effects could have impacted REM sleep in our study and contributed to the lack of significant heritability for this stage duration; however, these recordings were conducted in people's homes, not in a laboratory, which would reduce first‐night effects.

There is limited work on the heritability of WASO based on PSG. An actigraphy study of 132, 12‐year‐olds suggested that additive genetic factors contribute to 57% of the variance in WASO (Sletten et al., 2013). A study using two consecutive nights of sleep EEG reported a moderate genetic influence (32%) on WASO in 60 healthy adolescents (Rusterholz et al., 2018). In adults, an actigraphy‐based twin study (195 participants from 45 MZ and 50 MZ twin pairs) found a more modest heritability estimate of 26% (Gehrman et al., 2019). Thus, the data presented here, from 648 PSG recordings, represent a significant advance in statistical power. Some factors could explain the contradiction between these studies and ours, where no significant heritability was observed. Firstly, the other studies are based on actigraphy data, where WASO is computed indirectly based primarily on sustained nocturnal activity. By contrast, with PSG, WASO is calculated through actual time‐resolved physiological evidence of wakefulness (Marino et al., 2013). On the other hand, actigraphy‐derived WASO is based on measures collected over multiple consecutive nights of normal sleep. By contrast, the data in the current study are based on a single night of PSG recording without prior habituation, which makes it likely that the data collection itself may have contributed to the variability of WASO. WASO is one of the few sleep parameters shown to have a high degree of variability in a multi‐night ambulatory sleep monitoring study, indicating that this may not provide reliable data if only evaluated on a single night of sleep recording (Chouraki et al., 2023). Furthermore, we need to consider the mean age of our sample. It has been established that both WASO and AHI increase significantly with age (Moraes et al., 2014). Therefore, AHI may have contributed to the variability of WASO found in the current sample, impacting its heritability.

Previous literature on the heritability of sleep duration relies on twin studies. Importantly, there has been little agreement regarding how much sleep duration depends on genetic factors. The heritable component of sleep duration is moderated by age, being lowest in infancy and young adulthood, highest in adolescence, and decreased in adulthood, reaching 42%–45% on average. When examining only objectively measured sleep using actigraphy, a significant heritability estimate of sleep duration between 49% and 65% has been reported (Kocevska et al., 2021). As highlighted by Gehrman et al. (2019), actigraphy studies are important because this method offers an unobtrusive way to account for the high variability in sleep patterns by allowing multiple days of data collection; however, actigraphy cannot measure sleep stages, which requires PSG. PSG studies in twins are scarce, and conclusions are highly variable, ranging from an absence of significant genetic contribution in a sample of adolescents (Rusterholz et al., 2018) to an estimate of 27% of the variability in sleep duration being genetically controlled (Kocevska et al., 2021). In addition to the possible difference in the physiological nature of the measurement between assessment methods, PSG has an additional particularity that could have impacted the heritability estimates of sleep duration – the first‐night effect (Kocevska et al., 2021). Our experimental design comprised a single‐night ambulatory PSG; thus, it did not account for the first‐night effect (Agnew et al., 1966). However, the first‐night effect is considered a consequence of both sleeping in a novel environment (the laboratory) as well as wearing the PSG equipment. Our study minimized this by recording PSG in people's homes. Moreover, our participants represented a wide variety of ages. However, including age as a covariate in our model did not change the heritability estimate (*h* ^2^ = 18%).

The heritability of obstructive sleep apnea has previously been determined in a sample of the BHS cohort based on a portable type 3 PSG study (de Paula et al., 2016). The *h* ^2^ value for the adjusted model in the current study (0.27 ± 0.10, *p* = 0.001) replicates the previous report and confirms its validity as a covariate in our models. Overall, the addition of AHI as a covariate resulted in a negative impact on the significant estimates, thus demonstrating that AHI plays a role in the proportion of variance of sleep architecture.

One evident strength of our study is its use of extended families, which provides a higher variability of shared genotypes in a sizable sample of 684 participants evaluated objectively through PSG. In addition, heritability values are often inflated in twin studies. When extended families are studied, the amount of overestimation due to common environmental effects is less substantial (Kim et al., 2015). Another important aspect of our study is the mixed‐ancestry origin of our sample. Previously reported data on the heritability of sleep stages were derived from a population of European descent living in Europe. By contrast, the Baependi cohort study comprised an admixed sample of European, African and Native American ancestries living in South America (Beijamini et al., 2016). The availability of sleep cohorts from around the world is essential to providing the means of comparison to help understand sources of variation between populations, including cultural, environmental and genetic factors (Almasy, 2022). The richness of our sample fills this gap in the literature. Heritability quantifies the overall phenotypic variation attributable to genetic factors, making it possible to assess the importance of genes on the variation of traits within and across populations (Mayhew & Meyre, 2017). Indeed, multiple genetic variants have been associated with sleep traits through multiple experimental approaches, such as candidate gene studies and genome‐wide association studies (GWAS) (Madrid‐Valero & Gregory, 2023). Several genetic studies of sleep duration, published over several years, have been summarized in a recent review (Lane et al., 2023). Earlier, single candidate gene studies focusing on clock genes (*CLOCK*, *DEC2*) have been followed by increasingly extensive GWAS analysis combining self‐reported and objectively measured sleep duration. The strongest and most reproducible association has been at the locus for the developmental transcription factor *PAX8* locus (Lane et al., 2023). Thus, a substantial amount is already known about the genes in which polymorphisms contribute to the heritability we report.

The most well‐characterized single polymorphism relevant to sleep architecture is the coding‐region variable number tandem repeat (VNTR) polymorphism in the *PER3* gene (rs57875989), which has been shown to have a profound effect on sleep homeostasis, including duration of REM‐ and NREM sleep (Viola et al., 2007). No published GWAS are currently available for the duration of sleep stages, owing to the relatively limited number of individuals who have undergone both PSG and genome‐wide genotyping in multiple laboratories worldwide. Both the intriguing effects of the *PER3* polymorphism and the confirmation reported here that the duration of NREM sleep stages is heritable suggest the value of including genome‐wide genotyping in PSG studies and pooling data from different studies into future GWAS analysis.

Although unique in measuring sleep objectively through ambulatory PSG in a large family‐based sample, our study has some limitations. The ambulatory assessment of sleep provides a high ecological validity with respect to the participants' natural sleep patterns. However, this also means that it did not control for social and environmental conditions, such as light, noise, temperature and co‐sleeping, which will have effects on factors such as sleep latency, for instance, which would not have been present under laboratory conditions. Moreover, the single‐night design did not account for the first‐night effect, an aspect considered by previous authors (Ambrosius et al., 2008; Kocevska et al., 2021), which is particularly important because the expression of sleep stages shows a significant within‐subject variation, despite the already reported high stability of EEG across multiple sleep recordings (Buckelmüller et al., 2006). However, it is important to highlight that the first‐night effect refers not only to the PSG recording itself but also to sleeping in a different environment (e.g. laboratory or clinic), and the participants in this study slept in their homes.

In summary, we have demonstrated that additive genetic factors contribute to a significant fraction of the variance in the TST and duration of NREM sleep stages. This demonstrates the potential value in pooling data from genotyped individuals for collaborative GWAS.

## AUTHOR CONTRIBUTIONS

**Mario Leocadio‐Miguel:** Formal analysis; writing – original draft; writing – review and editing; methodology. **Tâmara P. Taporoski:** Conceptualization; methodology; data curation; investigation; project administration; writing – review and editing; software; writing – original draft. **Felipe Beijamini:** Conceptualization; methodology; software; investigation; writing – review and editing. **Francieli S. Ruiz:** Writing – review and editing; investigation. **Andrea R. V. R. Horimoto:** Software; formal analysis. **Alexandre C. Pereira:** Writing – review and editing; conceptualization; project administration. **Kristen L. Knutson:** Funding acquisition; conceptualization; methodology; investigation; data curation; supervision; project administration; writing – review and editing. **Malcolm von Schantz:** Funding acquisition; writing – original draft; conceptualization.

## CONFLICT OF INTEREST STATEMENT

Financial disclosure: this work was supported by National Institutes of Health grant 1R01HL141881. The authors have no other financial disclosures. Non‐financial disclosure: none.

## IRB STATEMENT

The protocol for this study conformed to international ethics standards based on the Declaration of Helsinki. It was approved by the local ethics committee (Hospital das Clínicas, University of São Paulo, Brazil, number 0494/10). Each volunteer provided informed consent before participation.

## Data Availability

The data that support the findings of this study are available from the corresponding author upon reasonable request.
